# Low-Cost Portable Sensor Node for Gas and Chemical Leak Detection with Kalman-Filtering-Based UWB Localization

**DOI:** 10.3390/s26102921

**Published:** 2026-05-07

**Authors:** Mohammed Faeik Ruzaij Al-Okby, Thomas Roddelkopf, Kerstin Thurow

**Affiliations:** 1 Center for Life Science Automation (Celisca), University of Rostock, 18119 Rostock, Germany; kerstin.thurow@celisca.de; 2 Technical Institute of Babylon, Al-Furat Al-Awsat Technical University (ATU), Kufa 54003, Iraq; 3 Institute of Automation, University of Rostock, 18119 Rostock, Germany; thomas.roddelkopf@celisca.de

**Keywords:** sensor node, hazardous gases, harmful gases, volatile organic compounds (VOCs), ambient monitoring, environmental monitoring, gas sensors, Kalman filters (KF), extended Kalman filter (EKF), unscented Kalman filter (UKF)

## Abstract

The work environment in automated laboratories and industrial sites exposes workers to the risks associated with chemical gas and vapor leaks caused by unforeseen incidents. Such leaks may result in severe health hazards as well as damage to equipment or infrastructure at the leak site. Therefore, the development of systems capable of early detection and highly accurate localization of chemical leaks is of high importance for occupational safety. In this work, a low-cost, portable sensor node based on the Internet of Things (IoT) is proposed for the detection and localization of gas and chemical leaks in indoor environments. The sensor node features a modular design that enables flexible integration and replacement of gas and environmental sensors depending on the target application. In addition, the system includes an ultra-wideband (UWB)-based positioning and tracking unit, allowing operation across multiple indoor zones. The main contribution of this work lies in the combined integration of (i) multi-sensor-based environmental event detection and prediction and (ii) high-precision location within a dynamic multi-zone tracking architecture. The system automatically selects the most relevant anchors in each zone and applies trilateration and least-squares estimation, enhanced by Kalman filtering techniques. In particular, an extended Kalman filter (EKF) and an unscented Kalman filter (UKF) are employed, with sensor fusion incorporating inertial measurement unit (IMU) data to mitigate the effects of on-line-of-sight (NLoS) conditions and signal degradation caused by obstacles. Experimental results demonstrate that both the EKF and UKF significantly reduce positioning errors and improve tracking stability compared to baseline methods under challenging indoor conditions. The UKF shows superior performance in highly nonlinear scenarios. A quantitative evaluation using manually surveyed reference points showed that the UKF achieved the best overall performance, with a mean error of 39.72 cm and an RMSE of 43.03 cm. These findings confirm the effectiveness of Kalman filter-based sensor fusion for reliable indoor positioning and highlight the suitability of the proposed system for real-time safety monitoring applications.

## 1. Introduction

In recent years, increasing attention has been paid to the monitoring of indoor air quality and airborne chemical pollution due to their impact on human health, safety, and productivity. This is particularly relevant in automated laboratories and industrial environments, where hazardous chemicals and gases are routinely handled. Accidental leaks of such substances may lead to severe health risks, environmental contamination, and damage to infrastructure, highlighting the need for reliable monitoring and early warning systems. In addition, increasing regulatory and industrial environments further emphasize the importance of continuous and intelligent environmental monitoring solutions.

Traditional environmental monitoring systems typically rely on high-precision equipment, which is often complex and expensive, limiting their scalability and widespread deployment. In contrast, recent research in this field has focused on the development of low-cost sensor networks capable of real-time air quality monitoring and anomaly detection. These systems commonly integrate gas sensors, particulate matter sensors, and environmental sensors to identify indoor pollution sources and detect abnormal environmental events. Sensor nodes have thus become key components of smart building monitoring and control systems, enabling continuous observation and early detection of hazardous conditions, such as chemical leaks, smoke, or fire.

Driven by advances in sensor technology and communication electronics, modern sensor nodes have evolved from simple data acquisition units into intelligent systems capable of multi-sensor data processing, wireless communications, and integration with Internet of Things (IoT) and edge computing frameworks. This evolution enables not only real-time monitoring but also local data processing and decision-making, reducing latency and dependence on centralized infrastructure. More recently, the increasing use of mobile robots and autonomous platforms in laboratories and industrial environments has created a demand for mobile sensor nodes that can detect hazardous substances and simultaneously determine their spatial location. Accurate localization of such events is essential for effective risk mitigation and targeted intervention, particularly in dynamic or large-scale indoor environments [1,2,3].

Baseline deviation analysis is widely used in environmental monitoring systems, where real-time measurements are compared against reference values to detect any anomalies. For example, elevated levels of volatile organic compounds (VOC), particulate matter (PM), or carbon dioxide equivalent (eCO_2_) may indicate chemical leaks, smoke generation, or combustion processes. Due to its computational efficiency, this approach is well-suited for real-time applications [4,5]. In addition, rule-based event detection frameworks have widely been employed to classify environmental conditions based on threshold exceedances and temporal trends, enabling the identification of events such as smoke emissions, chemical emissions, or fire incidents [6,7,8].

Several studies have addressed the development of sensor systems and localization approaches in indoor environments. Ge et al. proposed a machine learning-based indoor localization system that utilizes environmental and temporal sensor data, such as temperature, humidity, and light intensity, to classify indoor locations using algorithms including Random Forest, support vector machine, K-nearest neighbor, Decision Tree, and Adaptive Boosting [9].

Tan et al. introduced a magnetic induction-based localization system designed for environments with limited line-of-sight (LoS), demonstrating improved robustness in complex indoor scenarios [10].

Pham et al. developed a sensor fusion approach combining passive infrared sensor (PIR) and inertial measurement units (IMU) with particle filtering for indoor human localization, achieving high positioning accuracy [11].

Furthermore, LiDAR-based approaches have been explored for simultaneous localization and mapping (SLAM), providing high accuracy but typically at a higher cost and system complexity [12]. In the context of gas detection, Kumar et al. proposed an IoT-based system integrating gas sensor arrays with machine learning (ML) techniques for hazardous gas identification, achieving high classification accuracy in a controlled environment [13].

Recent studies have also explored advanced gas-sensing strategies for environmental and hazardous substance monitoring. In particular, plasmonic and material-engineered sensing approaches have demonstrated high sensitivity under controlled measurement conditions. However, such solutions typically rely on specialized sensing structures and more complex instrumentation, which may limit their direct integration into compact, low-cost, and mobile sensor platforms [14].

Other recent works have further highlighted the importance of accurate gas- and air-quality monitoring in safety-oriented applications. These studies confirm the increasing demand for reliable detection of hazardous environmental conditions. However, their primary focus remains on sensing performance itself, whereas the present work emphasizes the joint integration of environmental event detection and real-time indoor localization within a portable embedded architecture [15].

Beyond the co-existence of sensing and positioning modules, an important research gap remains in the interaction mechanism between these subsystems. In many existing implementations, environmental sensing and localization are treated as loosely connected processes, where detection results and positioning outputs are combined only at a superficial level. This often neglects critical aspects such as temporal alignment between sensor measurements and position estimates, as well as the potential for mutual awareness between environmental anomalies and localization uncertainties. For instance, sudden changes in environmental sensor readings caused by an event may coincide with reduced positioning accuracy under non-line-of-sight (NLoS) conditions, highlighting the need for a tighter coupling between environmental event detection and localization, rather than independent processing. Addressing this interaction requires deeper system-level integration, where sensor and positioning data are processed within a unified processing framework that supports consistent spatial and temporal data interpretation. This goes beyond hardware-level integration and enables coherent spatiotemporal alignment of environmental measurements and position estimates [16,17,18].

Despite these advances, existing approaches typically focus either on environmental sensing or on localization, while only a limited number of systems address the combined problem of real-time hazard detection and accurate indoor positioning in a unified low-cost architecture. In addition, many localization approaches suffer from reduced accuracy in indoor environments due to non-line-of-sight (NLoS) conditions and signal attenuation caused by obstacles such as walls and laboratory equipment. Moreover, the integration of reliable event classification with robust localization remains a significant challenge, particularly in dynamic and cluttered indoor settings.

To address these challenges, this work proposes a low-cost, portable sensor node that integrates multi-sensor-based environmental monitoring with high-precision indoor localization. Unlike many existing systems that treat sensing and localization as loosely coupled processes, the proposed approach emphasizes coordinated operation within a unified processing framework. This enables consistent spatiotemporal interpretation of environmental events, ensuring that sensing and localization data are jointly interpreted in both spatial and temporal domains.

The main contributions of this work can be summarized as follows:

- Development of a modular IoT-based sensor node for detecting hazardous gases, particulate matter, and environmental parameters;
- Design of a dynamic multi-zone indoor tracking system based on ultra-wideband (UWB) technology with adaptive anchor selection;
- Implementation and comparison of Kalman filter-based localization approaches (KF, EKF, and UKF), including sensor fusion with IMU data for improved robustness under NLoS conditions;
- Development of a rule-based multi-sensor environmental event detection framework for classifying VOC leaks, smoke, and fire events.

The remainder of this paper is organized as follows. Section 2 describes “Methods and Materials”, including the hardware and software components and system architecture. Section 3, “Work Description” presents the system design and the implemented algorithms. Section 4, “Experimental Tests and Results”, discusses the experimental setup and results. Section 5, “Discussion and Conclusions”, provides a detailed discussion of the findings, and concludes the paper and outlines directions for the future.

## 2. Materials and Methods

The proposed system consists of two main components: (i) the hardware platform and (ii) the monitoring software. The hardware comprises a prototype sensor node equipped with multiple environmental sensors and a positioning unit. The software is responsible for data processing, visualization, and event detection.

**Figure 1:**
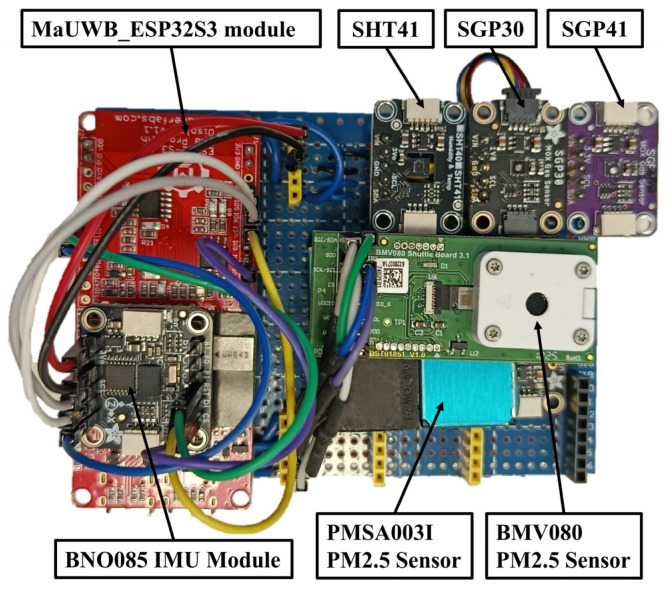
Top view of the components of the sensor node used.

The sensor node is designed as a modular platform that integrates different types of sensors for measuring gas concentrations, particulate matter (PM), temperature, and relative humidity. In addition, the sensor node incorporates an ultrawideband (UWB) tracking unit that operates in conjunction with multiple fixed anchors deployed within the building. The following subsections describe the individual system components in detail. Figure 1 illustrates the sensor node used in this work.

### 2.1. UWB Module

Ultra-wideband (UWB) technology forms the backbone of the positioning and tracking system, enabling precise indoor localization in real-time. UWB units can be deployed as mobile tags or carried by laboratory personnel, and can also be integrated into robotic platforms or autonomous systems operating in hazardous environments.

In this work, the MaUWB_ESP32S3 (Makerfabs, Shenzhen, China) was selected for system implementation. The module integrates several functional units. It is based on the DWM3000 transceiver (Qorvo, Greensboro, NC, USA), which provides key functionalities such as an integrated ceramic antenna, a real-time clock, RF front-end, and power management system. The transceiver supports two-way ranging (TWR) as well as time difference of arrival (TDoA) methods for distance estimation.

**Figure 2:**
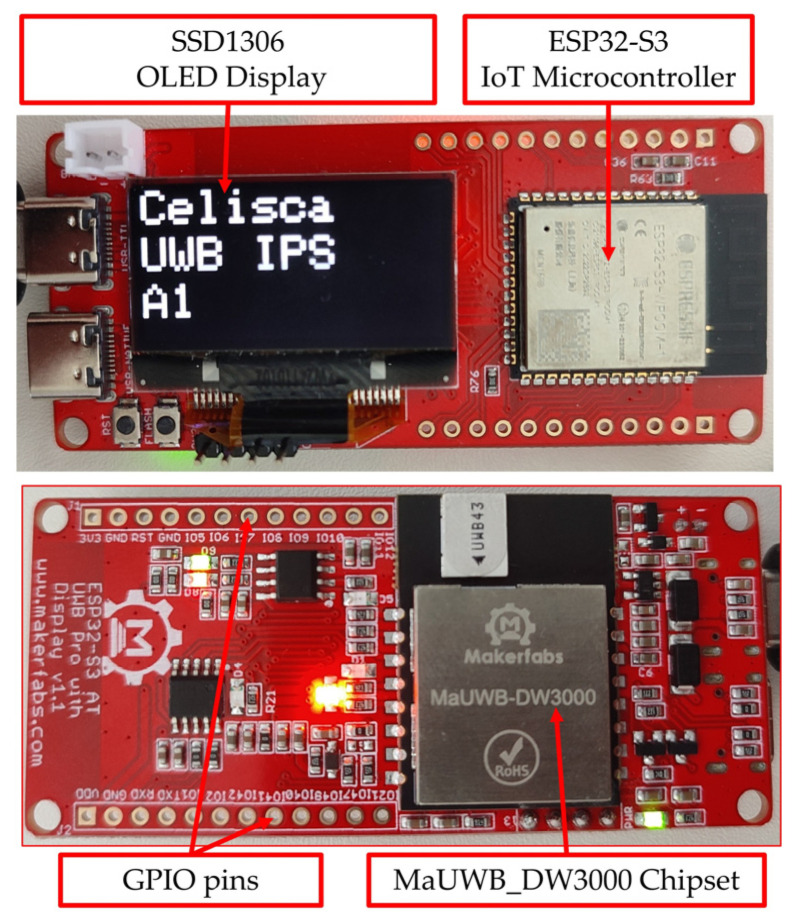
MaUWB_ESP32S3 UWB module.

The DWM3000 is controlled via AT commands using an integrated STM32F103RCT6 microcontroller (STMicroelectronics, Geneva, Switzerland), which allows low-level access to internal registers and configuration parameters. In addition, the module included an ESP32-S3 IoT microcontroller (Espressif Systems, Shanghai, China), which provided Wi-Fi and Bluetooth 5 connectivity. In the proposed system, the ESP32-S3 is used for processing UWB data and readings from various sensors, as well as for transmitting data to the monitoring and tracking server. The module further includes a 1.3-inch OLED display for local visualization and a set of input/output interfaces for sensor integration. Owing to its functionality and relatively low cost (approximately €50), the module represents a suitable solution for real-time indoor tracking applications. Figure 2 shows the UWB module used.

### 2.2. Gas and Environmental Sensors

To enable a reliable detection of chemical leaks, smoke, and fire-related events, the sensor node integrates multiple gas and environmental sensors. The SGP41 gas sensor (Sensirion AG, Stafa, Switzerland) is used to measure the volatile organic compound (VOC) index and the Nitrogen oxides (NO_x_) index. Both indices are unitless and range from 0 to 500, representing air quality levels from excellent to poor. These parameters provide important indicators for the detection of chemical leaks (VOC index) and combustion-related events (NO_x_ index). In addition, the SGP30 sensor (Sensirion AG, Stafa, Switzerland) is used to measure total VOC (TVOC) concentration and the carbon dioxide equivalent (eCO_2_) concentration [19,20].

Environmental conditions are further monitored using the SHT41 sensor (Sensirion AG, Stafa, Switzerland), which measures temperature and related humidity in the operational ranges of −40 to 125 °C and 0 to 100% RH, respectively. Rapid changes in temperature and humidity can indicate abnormal conditions such as fire or smoke. However, these parameters alone are insufficient for reliable detection, as they may also be influenced by other environmental factors. Therefore, their integration with gas and particulate matter sensors is essential to improve the detection robustness [21].

Two particulate matter sensors are employed to measure PM1 (1-micron diameter), PM2.5 (2.5-micron diameter), and PM10 (10-micron diameter) concentrations in the air: the PMSA003I (Nanchang Panteng Technology Co., Ltd., Nanchang, China) and the BMV080 (Bosch Sensortec, Reutlingen, Germany). The BMV080 is a compact sensor with a measurement range of 0 to 1000 μg/m^3^ and a resolution of 1 μg/m^3^, which makes it well-suited for portable applications. In addition, it can be hosted with any processor with an I2C or SPI bus [22]. The PMSA003I operated based on the laser scattering principle and communicates via an I^2^C interface [23].

### 2.3. BNO085 Inertial Measurement Unit

The BNO085 (CEVA Technologies, Inc., Rockville, MD, USA) inertial measurement unit (IMU) integrates a triaxial accelerometer, magnetometer, and gyroscope, providing nine degrees of freedom for the inertial measurement unit. These sensors are coupled with an embedded ARM Cortex M0 processor running the Hillcrest SH-2 sensor fusion firmware (Hillcrest Labs, Inc., Rockville, MD, USA). The IMS provides processed motion data, including yaw, pitch, and roll angles, quaternion, rotation vector, linear acceleration, and gravity vectors. In this work, IMU data are used to support the localization process by enabling sensor fusion with UWB measurements. Specifically, the IMU contributes to mitigating the effects of signal degradation and non-line-of-sight (NLOS) conditions, which commonly occur in indoor environments due to obstacles such as walls and equipment. The integration of IMU data with UWB measurements is performed within the unscented Kalman filter (UKF) framework described in Section 3 [24]. Table 1 summarizes the main sensors and hardware components integrated into the proposed system.

## 3. Work Description

The current project aims to design a low-cost, indoor chemical leak detection system with multi-tag, multi-zone tracking and location capabilities, utilizing commercially available components. It is important to note that the algorithms employed in this work are based on established methods. However, their integration, coordination, and adaptation within a dynamic multi-zone indoor environment represent the main contribution of the proposed framework. The system architecture consists of fixed anchors that continuously transmit UWB signals. These signals are received by a mobile tag, which calculates the distances from the eight nearest anchors in the operating environment and wirelessly transmits the sensor array data via Wi-Fi to a monitoring server. The moving tag sends data in the form of five JSON arrays, representing the tag number, distances from the nearest eight anchors, anchor numbers, environmental sensor data, and inertial measurement unit data. The transmission uses the UDP protocol to ensure high-speed, uninterrupted data flow along the tracking path.

On the monitoring server side, a Python 3.12 program receives, sorts, and processes the incoming data, applying algorithms to determine the tag positions and sensor readings with an initial guess of the expected environmental event based on the measurements.

The main tracking tasks of the monitoring program are summarized as follows:

1. Continuously receive UWB data via UDP for each tag.
2. For each tag,
  - Identify the active room (zone/room) based on the distances to anchors in the received UDP message.
  - Select the top three anchors (usually the closest/nearest within the room).
  - Calculate an initial position using Least Squares (LSQ) (three anchors).
  - Update smoothing/tracking filters (KF/UKF/EKF) using appropriate measurements.
3. Map the results:
  - Display only the anchors within the active rooms.
  - Highlight the three anchors currently in use.
  - Color tag points according to filter type.
4. Calculate the dynamic GDOP for active room(s) based on selected anchors and current tag position.
5. Store the results in a log file for analysis and performance evaluation.

The following sub-sections will describe the main algorithms in the monitoring and tracking program in detail.

### 3.1. Multi-Zone Room Selection

The monitoring program dynamically selects the active room/zone for each tag, regardless of the number of tags or zones. It does not assume a fixed room; instead, the active room is determined by calculating a weight score based on anchors that appeared in the received message. The anchors are predefined and operate in a single room only.

The active room is selected by summing the inverse values of the distances of anchors in each room:

(1) Active Rome= 1/(Range+ℇ)

where the ε value is a small amount, 0.001, and provides additional protection against division by zero when the distance is very small, which is a rare occurrence.

**Figure 3:**
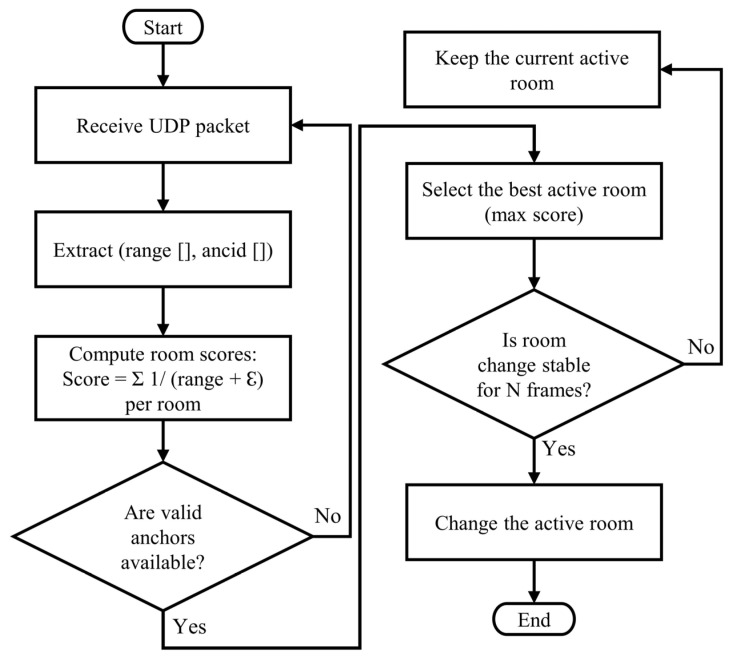
Flowchart of the active room selection algorithm.

Hysteresis is applied to avoid “flickering” of a room or zone when a tag crosses boundaries. The system only switches the active zone if the new room achieves the highest score in consecutive frames (e.g., 3). This process ensures the selection of the three closest anchors in the room or zone for localization. Figure 3 shows a flowchart of the active room/zone selection process. The proposed weighting and hysteresis mechanism is designed to ensure stable zone selection during transitions between adjacent rooms, where signal conditions and anchor visibility may change rapidly.

### 3.2. Anchor Selection

After defining the room, three anchors are selected for trilateration.

1. Valid measurements are collected for each anchor. If fewer than eight anchors are active, missing distances are replaced with (0) for distance and missing anchor numbers with (−1).
2. Anchors belonging to the selected room are preferred.
3. If fewer than three anchors are available, the system falls back to the nearest three anchors to prevent the program from crashing or obtaining inaccurate results during trilateration.
4. The selected anchors are stored in the active anchors matrix for the current tag and are used for
  - LSQ/KF calculations;
  - UKF/EKF ranges;
  - Map highlights;
  - Dynamic GDOP computation.

Dynamic GDOP is calculated per frame based on the current tag position. If multiple tags occupy the same room, the average GDOP for the room is used, which causes the GDOP index to change as the tag moves from one area to another.

### 3.3. Least Squares Algorithm (LSQ)

The Least Squares Estimation method (LSQ) estimated tag coordinates (*x*, *y*) by selecting three distance measurements from the three nearest anchors with predefined coordinates within the monitoring program (*x_i_*, *y_i_*) and real-time distance measurements (di). The model is based on the circle equation for each anchor:

(2) $$d_{i}^{2}={\left(x-x_{i}\right)}^{2}+{\left(y-y_{i}\right)}^{2}$$

Subtracting the first anchor equation from the others eliminates the quadratic terms (*x^2^*, *y^2^*), resulting in a linear system:

$$\mathrm{Ap}=b,p=\left[\begin{array}{c} x\\ y \end{array}\right]\;=\left[\begin{array}{cc} 2(x_{2}-x_{1}) & 2(y_{2}-y_{1})\\ 2(x_{3}-x_{1}) & 2(y_{3}-y_{1}) \end{array}\right]\;b=\left[\begin{array}{c} d_{1}^{2}-d_{2}^{2}{+x}_{2}^{2}+y_{2}^{2}-x_{1}^{2}-y_{1}^{2}\\ d_{1}^{2}-d_{3}^{2}{+x}_{3}^{2}+y_{3}^{2}-x_{1}^{2}-y_{1}^{2} \end{array}\right]$$

The “*p*” value (tag coordinates) is estimated by solving the LSQ:

(3) $$p={{(A}^{T}A)}^{-1}A^{T}b$$

when (*A^T^ A*) is reversible, which is implemented by the tracking program code via the numpy.linalg.lstsq function, the resulting coordinates are measurements for subsequent Kalman filtering [25].

### 3.4. Linear Kalman Filter (KF)

A linear Kalman filter was used to smooth and track the position of the moving tag using a four-state model, x = [x, y, v_x_, v_y_]^T^, by assuming constant velocity. With a time step of dt, the transition equation is

$$x_{k|k-1}={F\;x}_{k-1|k-1},F=\left[\begin{array}{cccc} 1 & 0 & dt & 0\\ 0 & 1 & 0 & dt\\ 0 & 0 & 1 & 0\\ 0 & 0 & 0 & 1 \end{array}\right],$$

Position is measured only from the LSQ in the monitoring software via

$$z_{k}={\mathrm{Hx}}_{k}+v_{k},H=\left[\begin{array}{cccc} 1 & 0 & 0 & 0\\ 0 & 1 & 0 & 0 \end{array}\right],$$

where *v*_*k*_∼*N*(0,*R*). KF undergoes two standard stages:

The prediction

P_k∣k−1_ = FP_k−1∣k−1_F^T^ + Q, (4)

And the update

K_k_ = P_k∣k−1_H^T^(HP_k∣k−1_H^T^ + R)^−1^,

x_k∣k_ = x_k∣k−1_ + K_k_(z_k_ − Hx_k∣k−1_),  

P_k∣k_ = (I − K_k_H)P_k∣k−1_                   

The KF provides a smooth estimate of tag position (P) and tag speed (v). The instantaneous tag speed is calculated based on the velocity components produced by the Kalman filter using the equation v = √(*v*_*x*_^2^ + *v*_*y*_^2^). The adopted linear Kalman filtering framework follows the standard discrete-time formulation widely used in tracking applications [26,27].

### 3.5. Extended Kalman Filter (EKF)

The EKF handles the nonlinear relationship between UWB ranges and tag position, accounting for NLoS and multipath propagation. Instead of using the initial coordinates in LSQ, the moving tag coordinates are calculated using distance measurements from the three selected anchors in the effective region. When the measurement is the range measurement vector to the anchors *z* = [*r*1, *r*2, *r*3]^T^ and not the position directly, the system state is defined for the KF as *x* = [*x*, *y*, *v_x_*, *v_y_*]*^T^* with a linear motion model F (constant velocity). The nonlinear measurement function is

$$H_{i}(x)=\sqrt{{\left(x-a_{x,i}\right)}^{2}+{\left(y-a_{y,i}\right)}^{2}},$$

where *i* = 1, 2, 3,

z_k_ = h(x_k_) + v_k_.

The EKF linearizes this function using the Jacobian matrix:

(5) $$H_{k}={\left.\frac{\partial h(x)}{\partial x}\right|}_{x=x_{k|k-1}},$$

where the anchor row *i* is approximately equal to

$$\frac{\partial h_{i}}{\partial x}=\frac{x-a_{x,i}}{d_{i}},\frac{\partial h_{i}}{\partial y}=\frac{y-a_{y,i}}{d_{i}},\frac{\partial h_{i}}{\partial v_{x}}=0,\frac{\partial h_{i}}{\partial v_{y}}=0,$$

using

$$d_{i}\left(x\right)=\sqrt{{\left(x-a_{x,i}\right)}^{2}+{\left(y-a_{y,i}\right)}^{2}}+\varepsilon$$

where a small ε may be added in implementation to avoid division by zero to ensure numerical stability. Then, the same Kalman linear update equations are implemented, but using H_k_. The innovation is computed as

y_k_ = z_k_ − h(x_k∣k−1_),      

S_k_ = H_k_P_k∣k−1_H_k_^T^ + R,

K_k_ = P_k∣k−1_H_k_^T^Sk − 1  

In this way, EKF deals with the nonlinearity of UWB (distance) measurements through a local linear approximation around the expected state [28,29,30].

### 3.6. Unscented Kalman Filter (UKF)

The UKF is employed to estimate the tag position by fusing UWB range measurements with IMU data. Unlike the EKF, the UKF avoids explicit linearization by propagating a set of deterministically chosen sigma points through nonlinear system dynamics. The system state is defined as

$$x_{k}={\left[x_{k}{\;y}_{k}\;v_{x,y}\;v_{y,k}\right]}^{T}$$

where (x_k_, y_k_) represents the position and (v_x,k_, v_y,k)_ the velocity.

For a state of dimension nnn, a set of 2n + 1 sigma points is generated:

$$X_{0}=x,\;\;X_{i}=x+(\sqrt{(n+\lambda )P}{)}_{i},\;\;X_{i+n}=x-(\sqrt{(n+\lambda )P}{)}_{i},$$

where *i* = 1, …, *n*, with weights

$$W_{0}^{\left(m\right)}=\frac{\lambda }{n+\lambda },W_{0}^{\left(c\right)}=\frac{\lambda }{n+\lambda }+(1-\alpha^{2}+\beta ),W_{i}^{\left(m\right)}=W_{i}^{\left(c\right)}\frac{1}{2(n+\lambda )}$$

In contrast to a standard constant-velocity model, the proposed approach incorporates IMU acceleration as an external input to the motion model. The continuous-time dynamics are defined as

$$\bar{x}=v_{x},\bar{y}=v_{y},{\bar{v}}_{x}=a_{x},{\bar{v}}_{y}=a_{y}$$

After discretization, the state evolution becomes:

$$x_{k+1}=x_{k}+v_{x,k}∆t+\frac{1}{2}a_{x}{∆t}^{2}\;y_{k+1}=y_{k}+v_{y,k}∆t+\frac{1}{2}a_{y}{∆t}^{2}$$

$$v_{x,k+1}=v_{x,k}+a_{x}∆t\;v_{y,k+1}=v_{y,k}+a_{y}∆t$$

The acceleration components a_x_, a_y_ are derived from the IMU through

- Coordinate transformation (body → world frame):
  $$a^{w}=R_{\left(q\right)}a^{b}$$

- Low-pass filtering:
  $$a_{f}={\alpha a}_{f,prev}+{(1-\alpha )a}_{raw}$$

- Bias estimation (during quasi-static conditions):
  $$b=\left(1-\beta \right)b+\beta a_{f}$$
  $$a=a_{f}-b$$

Thus, the IMU contributes directly to the state transition function:

$$X_{i}^{k+1}=f(X_{i}^{k},a,∆t)$$

which improves short-term motion prediction (dead-reckoning).

The measurement vector consists of distances to anchors:

$$z_{k}=\left[\begin{array}{c} d_{1}\\ d_{2}\\ d_{3} \end{array}\right]$$

with

$$d_{j}=\sqrt{{\left(x-a_{x,j}\right)}^{2}+{\left(y-a_{y,j}\right)}^{2}}.$$

Each sigma point is projected into measurement space:

$$Z_{i}=h(X_{i}),z=h(x)=\left[\begin{array}{c} \sqrt{{\left(x-a_{x,1}\right)}^{2}+{\left(y-a_{y,1}\right)}^{2}}\\ \sqrt{{\left(x-a_{x,2}\right)}^{2}+{\left(y-a_{y,2}\right)}^{2}}\\ \sqrt{{\left(x-a_{x,3}\right)}^{2}+{\left(y-a_{y,3}\right)}^{2}} \end{array}\right],$$

The predicted measurement mean and covariance

$${\hat{z}}_{k}=\sum W_{i}^{\left(m\right)}Z_{i}\;P_{zz}=\sum W_{i}^{\left(c\right)}{(Z}_{i}-{\hat{z}}_{k}){{(Z}_{i}-{\hat{z}}_{k})}^{T}\;P_{xz}=\sum W_{i}^{\left(c\right)}{(X}_{i}-{\hat{x}}_{k}){{(Z}_{i}-{\hat{z}}_{k})}^{T}$$

The Kalman gain is derived using the unscented transform as $K=P_{xz}P_{zz}^{-1}$, and the state update is expressed as follows

$$x_{k|k}=x_{k|k-1}+K{(z}_{k}-{\hat{z}}_{k})\;P_{k|k}=P_{k|k-1}-KP_{zz}K^{T}$$

The proposed framework performs loosely coupled sensor fusion: IMU data are integrated in the prediction step as control inputs, and the UWB measurements are used in the update step for absolute position correction. This complementary structure enables

- IMU → smooth short-term motion (dead-reckoning);
- UWB → long-term drift correction.

Finally, the system performs integrated sensing and localization through a time-synchronized processing pipeline, where environmental measurements and localization estimates are aligned in real time. This enables direct spatial association of detected events with their corresponding positions, forming a unified spatiotemporal representation of the environment [31,32,33].

### 3.7. Environmental Events Analysis and Detection Algorithm

The environmental event analysis and detection algorithm employs a rule-based event prediction approach that transforms multi-sensor readings into a probabilistic description of the environmental condition, such as normal conditions, VOC leaks, smoke, or fire.

#### 3.7.1. Definition of Weights Used for Measured Factors

Decision-making in the detection and analysis algorithm relies on the weighting coefficients assigned to environmental sensor features relevant to a given event. The weights were determined based on a series of practical experiments and observations of the algorithm’s response under different scenarios. In particular, the impact of different weight configurations was evaluated with respect to decision accuracy and the avoidance of misclassification or false alarms. This considers the expected characteristics of each event type, including VOC leaks, smoke, and fire. Fixed weights were assigned to each feature-event combination and remain constant during operation, while being adjustable based on system performance if required. Higher weights were assigned to features strongly associated with a particular event (e.g., particulate matter for smoke and temperature for fire). In addition, negative weights were introduced to reduce the influence of misleading or non-discriminative signals (e.g., the effect of particulate matter alone on VOC events). Table 2 illustrates the weights used in the weighting functions.

#### 3.7.2. Feature Extraction

The process begins with real-time acquisition of environmental sensor data, including temperature, humidity, VOC index, NO_x_ index, total volatile organic compound (TVOC) concentration, carbon dioxide equivalent (eCO_2_), and particulate matter concentrations (PM2.5). Sensors used are PMSA003I and BMV080. The algorithm does not rely solely on raw sensor values; instead, it constructs a dynamic baseline for each variable under normal conditions and calculates the relative deviation from this baseline:

$${∆}_{r}\left(x\right)=\frac{x-b}{max(\left|b\right|,1)}$$

where *x* is the current measurement and *b* is the baseline. Absolute baseline values below 1 are ignored to prevent division by zero and avoid inflated deviations. This metric indicates how much the current measurement deviates from normal environmental conditions. Additionally, the algorithm calculates the time rates of change for each parameter to capture rapid variations that may be more indicative of an event than absolute values:

$$\dot{x}=\frac{x\left(t\right)-x(t-1)}{∆t}$$

Critical parameters include $\frac{d(TVOC)}{dt}$, $\frac{d(PM2.5)}{dt}$, $\frac{d(eCO2)}{dt}$, $\frac{d(T)}{dt}$, and $\frac{d(RH)}{dt}$.

The program creates weighted scores for each possible event type, combining multiple indicators into a single score for VOC leakage, smoke, and fire.

- VOC leaks are characterized by high TVOC concentrations and VOC index, with low particulate matter. The event score increases with Δr (TVOC) and Δr (VOC index) over time and decreases if fine particles rise significantly.
- Smoke is indicated primarily by a rapid increase in PM2.5 rate of change, with minor contribution from TVOC.
- Fire is considered a complex combustion event, with intensity increasing when multiple parameters rise simultaneously, including PM2.5, eCO_2_, and temperature over time.

The event scoring logic can be expressed as

$${Score}_{event}=\sum_{i}w_{i}f_{i}(x_{i},{\dot{x}}_{i},{∆}_{r}(x_{i}))$$

where *w*_i_ represents the experimental weights, *x*_I_ are measured variables (e.g., TVOC, PM2.5, or eCO_2_), ${\dot{x}}_{i}$ is the time derivative of *x_i_*, and *f_i_* incorporates both the relative deviation and the rate of change.

Each feature *f_i_* combines multiple components derived from sensor data:

$$f_{i}=w_{1}.{∆}_{r}\left(x_{i}\right)+w_{2}.{\dot{x}}_{i}+w_{3}.(x_{i}-b_{i})$$

where

- *w*_1_, *w*_2_, and *w*_3_ are weighting coefficients that determine the relative contribution of each feature;
- *w*_1_ corresponds to the weight of the relative change Δ_r_(x_i_);
- *w*_2_ corresponds to the weight of the rate of change ${\dot{x}}_{i}$;
- *w*_3_ corresponds to the weight of the absolute deviation from baseline (x_i_ − b_i_).

This formulation follows established principles in time-series anomaly detection, where multiple complementary features are combined to capture deviations from normal behavior [34,35,36].

An environmental event is identified when the resulting score surpasses a predefined threshold.

$${Score}_{event}>\tau_{e}$$

Here, τ denotes an empirically determined threshold specific to each event, derived from experimental observations. The index *e* indicates the category of the environmental event (such as a VOC leak, smoke, or fire). These thresholds are event-specific (VOC leak, smoke, and fire) and remain fixed during system operation.

For each event category, an independent score is calculated: ${Score}_{VOC},{Score}_{Smoke},{Score}_{Fire}$. The final decision is obtained by selecting the event with the highest score, provided that the required activation condition is satisfied. In cases of overlapping signals, priority is given to fire detection due to its critical safety relevance, followed by smoke detection, and finally VOC leakage.

To avoid rapid oscillation in event classification, the system employs a two-state time-stabilization mechanism:

1. The candidate state represents a potentially new event.
2. The current steady state represents the confirmed environmental condition.

The system transitions from steady state to candidate state only if the same candidate event is detected for a user-defined number of consecutive cycles. This allows the user to adjust sensitivity before final classification. Otherwise, the previous state remains unchanged. This logic reduces false alarms and prevents positive errors. Once confirmed, the system provides a visual warning indicating the event type, event color, approximate confidence level, and precise location of the detected event.

#### 3.7.3. Thresholds and Baseline Adaptation Strategy

The selected thresholds and weighting factors were determined empirically based on experimental observations under controlled indoor scenarios. In addition to the assessment mechanism described in the previous section, the proposed algorithm incorporates indicative threshold levels and an adaptive baseline update mechanism to ensure robust performance under varying environmental conditions. The detection process uses thresholds that can be adjusted based on user feedback and the baseline level of the operating environment. These thresholds are derived from empirical observations and typical indoor air quality ranges. The current threshold values are VOC index = 150 and NO_x_ index = 5, TVOC = 67 ppb, and PM2.5 level = 150 mg/L, which significantly exceed normal background conditions and act as trigger levels for event detection. However, these thresholds are not used as strict binary decision boundaries but rather as soft trigger conditions within the assessment framework, allowing gradual transitions instead of abrupt classifications. The baseline for each environmental parameter is initialized from the first valid measurements, requiring a warm-up period of approximately 5 min after the first operation of the sensor node to ensure the completion of the heating phase. After initialization, the baseline is continuously updated using a low-rate exponential moving average:

$$b_{t+1}=\left(1-\alpha \right)b_{t}+\alpha x_{t}$$

where α is a small smoothing coefficient (set to 0.01 in this work). This formulation enables slow adaptation to long-term environmental changes while maintaining sensitivity to transient events. Importantly, baseline updating is performed only under stable “normal” conditions and is temporarily suspended when abnormal events are detected. This prevents contamination of the reference baseline during hazardous situations and improves the robustness of anomaly detection.

## 4. Experimental Tests and Results

Two types of practical tests were conducted to evaluate the system’s performance. The first set of experiments assessed the environmental event analysis algorithm, focusing on the detection of smoke, fire, gas leaks, and chemical vapors. The second set evaluated the indoor tracking and positioning system under different Kalman filtering strategies, comparing their accuracy and robustness. The details of both tests are described in the following subsections.

### 4.1. Environmental Events Analysis and Detection Tests

These experiments targeted three types of environmental incidents expected in chemical handling facilities: chemical (VOC) leaks, smoke, and fire.

For the detection of chemical or VOC leaks, 99% ethanol was chosen to evaluate the algorithm’s performance. Very small volumes of ethanol (10, 30, 50, and 100 microliters) were used, placed in a Petri dish 1 m away from the sensor node.

**Figure 4:**
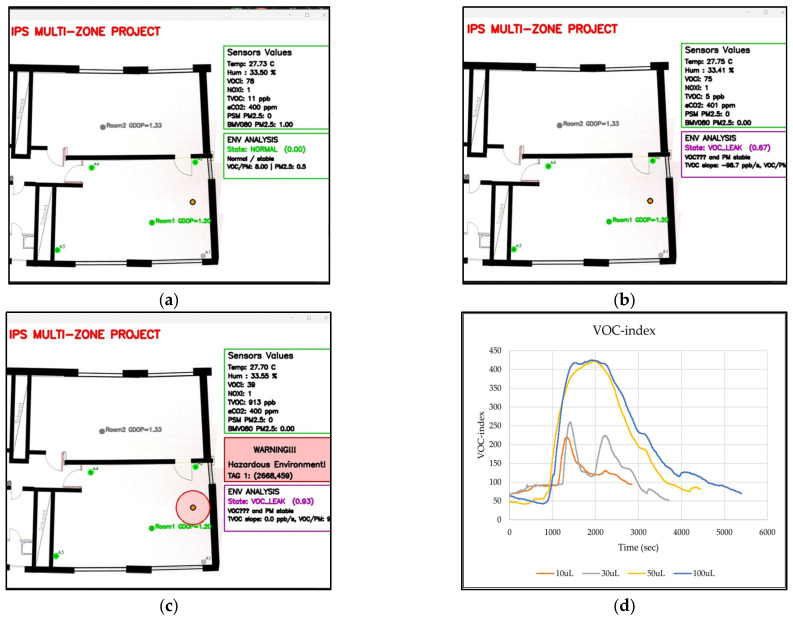
Screenshots of the monitoring program for VOC leak detection (**a**) before the sample is placed, (**b**) after the sample is placed and before reaching the alarm threshold, (**c**) after crossing the alarm threshold, and (**d**) the SGP41 sensor responses for the tested ethanol volumes.

The algorithm successfully detected VOC leakage for all tested samples. Figure 4a–c illustrates the monitoring program before and after the alarm threshold was reached, and Figure 4d shows the corresponding SGP41 sensors’ response.

**Figure 5:**
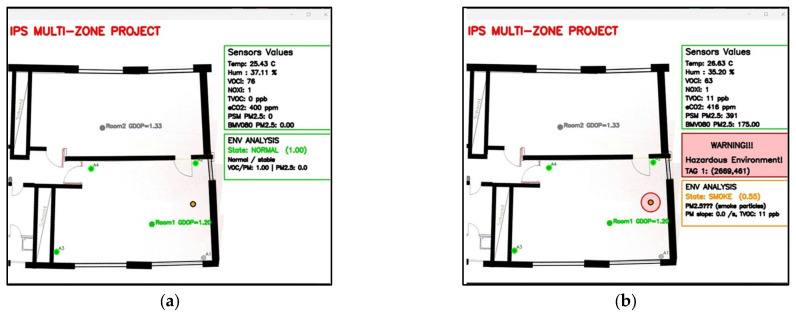
Screenshots of the monitoring program for smoke detection (**a**) before spraying the test spray, (**b**) after spraying the test spray.

To test the smoke event, a special type of spray, “LogiLink Rauchmelder” Test-Spray (2direct GmbH, Schalksmühle, Germany), designed for testing smoke alarms in homes, was used. The test was performed by spraying a sample of the aerosol from a distance of 1 m towards a sensor node at a fixed location. The test was repeated 10 times, and the algorithm succeeded in 8 out of 10 trials; two trials produced false-positive identifications. Figure 5 shows monitoring screenshots before and after spraying the test aerosol for smoke detection.

**Figure 6:**
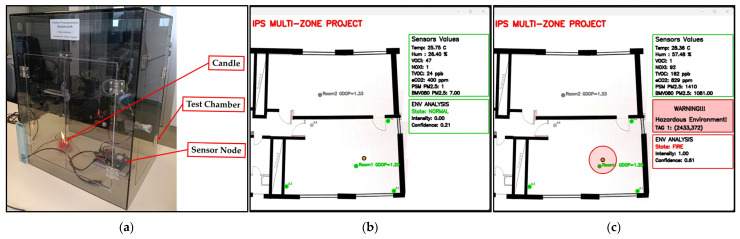
Testing for fire detection: (**a**) test chamber, screenshots of the monitoring program (**b**) before the fire, (**c**) after the fire.

Due to the presence of a fire-sensitive system in the laboratories, a special gas-testing chamber made of polyvinyl chloride (PVC) was used to completely cover the sensor node and prevent the smoke produced during the experiment from escaping. A candle was placed inside the chamber, and the sensor node test began by placing the sensor node outside, then lighting the candle, then inserting the sensor node inside the chamber, and monitoring the sensor node via the monitoring program. This experiment was repeated 10 times, and the detection result was correct for all ten tests. Figure 6a shows the test chamber; Figure 6b,c show a screenshot of the monitoring program before and after the fire was lit inside the test chamber.

### 4.2. Indoor Tracking and Positioning Tests with Kalman Filtering

**Figure 7:**
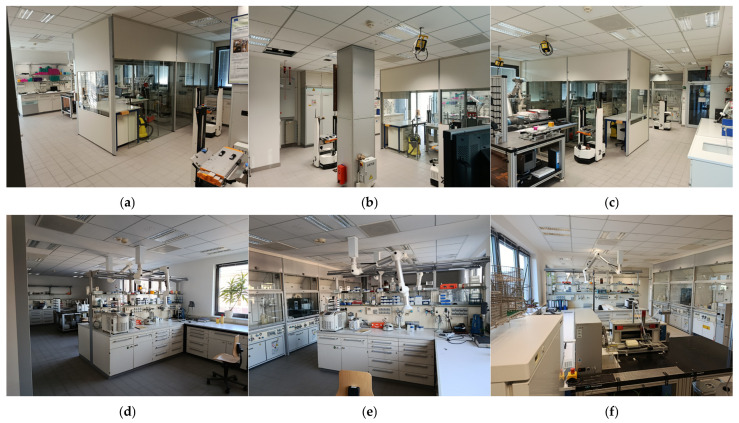
Test environments (**a**–**c**) Lab 1, and (**d**–**f**) Lab 2.

**Figure 8:**
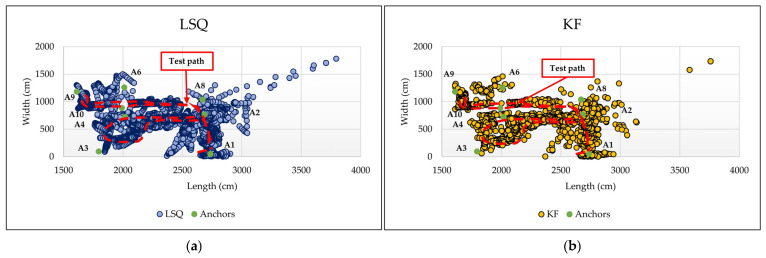

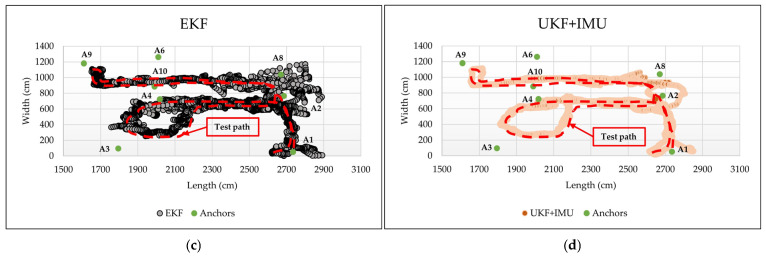
System tracking testing for movable tag in Lab. 1 and Lab. 2 using (**a**) least square, (**b**) Kalman filter, (**c**) extended Kalman filter, (**d**) unscented Kalman filter.

The objective of these experiments was to determine the optimal Kalman filter for improving positioning performance accuracy under prevalent no-line-of-sight (NLoS) conditions in indoor environments. The tracking system tests were conducted in two adjacent automated laboratories with numerous large obstacles obstructing UWB signal propagation. The laboratories are separated by a thick concrete wall and aluminum-framed glass doors that allow movement between them. Extensive laboratory furniture extends along the dividing wall, creating a challenging indoor environment (see Figure 7). Four anchors were installed in each laboratory at a height of two meters from the floor to mitigate the effects of NLoS. The actual test path was predefined and physically fixed on the floor across both laboratories (Room 1 and Room 2). The trajectory was designed to intentionally traverse different indoor conditions, including line-of-sight (LoS) and non-line-of-sight (NLoS) regions caused by walls, doors, and laboratory equipment. This setup enables a realistic evaluation of the tracking system under varying propagation conditions. The recorded results are stored on the monitoring server as log files containing the coordinate data for each algorithm or filter used for each moving marker. Figure 8 shows the test results for the tracking system: (a) using the least squares algorithm, (b) using the linear Kalman filter (KF), (c) using the extended Kalman filter (EKF), and (d) using the unscented Kalman filter (UKF).

### 4.3. Quantitative Localization Error Evaluation

To quantitatively evaluate the localization accuracy, 23 manually surveyed reference points were selected in Room 1 under representative LoS and NLoS conditions. For each reference point, the mobile tag was kept stationary, and the estimated coordinates obtained by LSQ, KF, EKF, and UKF were recorded over the measurement interval. The mean estimated position for each method was then computed, and the Euclidean localization error with respect to the reference coordinates was calculated as

$$e=\sqrt{{\left(x_{est}-x_{ref}\right)}^{2}+{\left(y_{est}-y_{ref}\right)}^{2}}$$

where $x_{ref},y_{ref}$ denotes the manually measured reference position, and ($x_{est},y_{est}$) represents the estimated tag position.

The quantitative results confirm the advantage of nonlinear filtering methods over baseline approaches. In particular, the UKF achieved the best overall performance, with a mean error of 39.72 cm and an RMSE of 43.03 cm, compared to 41.26 cm and 44.58 cm for EKF, 79.74 cm and 84.54 cm for KF, and 79.13 cm and 83.80 cm for LSQ. Relative to LSQ, the UKF reduced the mean localization error by approximately 49.8% and the RMSE by approximately 48.7%. Table 3 shows the quantitative localization error comparison for LSQ, KF, EKF, and UKF.

## 5. Discussion and Conclusions

The practical tests conducted on the proposed system provide strong evidence of its effectiveness in both environmental event detection and indoor tracking. The environmental event analysis algorithm demonstrated robust performance, achieving full detection of ethanol leaks and fire events, while smoke detection showed a slightly lower success rate with occasional misclassification. These results indicate that the algorithm can reliably identify hazardous events using real-time sensor data, while the integration of dynamic baselines and time-derivative analyses allows it to account for both absolute and rapid changes in environmental conditions. The use of a two-stage stabilization mechanism further reduces false alarms, ensuring that transient fluctuations in sensor readings do not trigger unwarranted alerts. In addition, the observed performance highlights the suitability of combining multiple low-cost sensors to achieve robust detection capabilities without relying on expensive analytical instrumentation, which is particularly relevant for scalable deployment scenarios.

The tracking and positioning system also exhibited promising results. The active room anchor selection algorithm effectively mitigates the influence of anchors from adjacent rooms, which could otherwise introduce errors due to signal propagation through walls. While the least squares and linear Kalman filter approaches provide baseline tracking performance, significant improvements were observed when nonlinear filtering techniques were applied. The extended Kalman filter (EKF) reduced errors caused by nonlinearity in UWB distance measurements, and the unscented Kalman filter (UKF) further enhanced accuracy and smoothness by fusing UWB and inertial measurement unit data. Even under challenging NLoS conditions, the UKF-based system maintained reliable positioning, highlighting the value of sensor fusion in indoor localization. In addition to the qualitative trajectory comparison shown in Figure 8, the quantitative evaluation based on 23 manually surveyed reference points confirms the superiority of the nonlinear filtering approaches over LSQ and KF. The UKF achieved the best overall performance, with a mean error of 39.72 cm and an RMSE of 43.03 cm, followed closely by the EKF. These results indicate that the improvement is not only visual, but also supported by objective numerical error metrics under representative indoor LoS/NLoS conditions. These results emphasize that incorporating motion dynamics and inertial information is essential for achieving stable localization in complex indoor environments.

Unlike simple post-processing approaches, the proposed system maintains a continuous spatiotemporal coupling between sensing and localization, enabling real-time mapping of environmental events. Although the current implementation does not employ fully joint probabilistic fusion, it provides a practical integration framework that combines motion-aware localization with environmental context. This work demonstrates that a low-cost system can simultaneously achieve environmental event detection and precise indoor tracking, offering real-time hazard identification and localization. From an application perspective, this approach is particularly beneficial for automated laboratories, smart industrial environments, and safety-critical infrastructures, where rapid and reliable response to hazardous events is required. The modular architecture of the proposed system further supports its adaptability and integration into existing IoT-based monitoring frameworks. For future research, expanding the detection tests to a broader range of volatile organic compounds and optimizing the algorithmic weighting of environmental factors will help to further enhance prediction accuracy. Additionally, while UKF improves resilience to NLoS errors, some residual positioning errors remain, suggesting the need for further refinement of the system architecture and filtering strategies, for example, through advanced fusion methods or adaptive filtering techniques. Overall, the presented approach provides a scalable and practical solution for chemical safety in laboratory and industrial environments, integrating predictive analytics and sensor-fusion localization in a cohesive framework. Future work will investigate tighter coupling between sensing and localization, including joint probabilistic estimation and cross-module feedback.

## Figures and Tables

**Table 1:** Overview of sensors and hardware components used in the proposed system.

Component Type	Model	Manufacturer	Measured Parameters	Measurement Range/Output	Interface
UWB Module	MaUWB_ESP32S3	Makerfabs	Distance, position (via UWB)	cm-level accuracy	UART/SPI
UWB	DWM3000	Qorvo	Time-of-flight	-	SPI
Microcontroller	ESP32-S3	Espressif Systems	Data processing, communication	-	Wi-Fi/BT
Gas Sensor	SGP41	Sensirion AG	VOC index, NO_x_ index	0–500 (index)	I^2^C
Gas Sensor	SGP30	Sensirion AG	TVOC, eCO_2_	TVOC: 0–60,000 ppb; eCO_2_: 400–60,000 ppm	I^2^C
Environmental Sensor	SHT41	Sensirion AG	Temperature, humidity	−40–125 °C; 0–100% RH	I^2^C
PM Sensor	PMSA003I	Plantower	PM_1_, PM_2.5_, PM_10_	0–1000 µg/m^3^	I^2^C
PM Sensor	BMV080	Bosch Sensortech	PM_2_, PM_2.5_, PM_10_	0–1000 µg/m^3^ (1 µg/m^3^ resolution)	I^2^C
IMU	BNO085	Bosch Sensortech/Hillcrest Laboratories	Acceleration, rotation, orientation	9-DOF output (quaternion, Euler angles)	I^2^C, SPI
Display	OLED (1.3″)		Visualization	128 × 64 pixels	I^2^C, SPI

**Table 2:** Event-dependent feature weighting scheme for multi-sensor fusion.

Event	Feature	Weight	Interpretation
VOC leak	TVOC change	2.5	primary indicator of VOC release
VOC leak	VOC index change	2.5	supporting gas-quality indicator
VOC leak	PM change	−1.5	suppresses confusion with smoke
Smoke	PM change	3.0	dominant smoke feature
Smoke	PM slope	0.06	fast smoke buildup
Smoke	TVOC change	0.4	secondary contribution
Fire	PM change	2.2	combustion particles
Fire	eCO_2_ change	2.2	combustion-related gas accumulation
Fire	Temperature change	3.0	strongest fire indicator
Fire	NO_x_ index change	0.8	secondary combustion-related cue

**Table 3:** Quantitative localization error comparison for LSQ, KF, EKF, and UKF.

Method	Mean Error (cm)	RMSE (cm)	Std (cm)	Max Error (cm)	95th Perc. (cm)	Median (cm)
LSQ	79.13	83.80	28.22	135.43	134.00	76.68
KF	79.74	84.54	28.69	135.24	132.71	76.18
EKF	41.26	44.58	17.27	79.72	69.52	40.44
UKF	39.72	43.03	16.93	76.86	65.11	38.56

## Data Availability

The data presented in this study are available on request from the corresponding author due to institutional restrictions and the use of proprietary software and resources belonging to our institute.
